# Effect of Primary Breast Surgery on Prognosis in Breast Cancer Patients Presenting with Isolated Bone Metastases

**DOI:** 10.3390/cancers18111760

**Published:** 2026-05-28

**Authors:** Abdulmunir Azizy, Izzet Dogan, Serap Yucel, Irmak Duru Subasi, Mustafa Bozkurt, Onur Dulgeroglu, Ali Arican, Ibrahim Yildiz, Cihan Uras

**Affiliations:** 1 Department of Medical Oncology, Istanbul University Institute of Oncology, Istanbul 34093, Turkey; munir.azizy@gmail.com; 2 Department of Medical Oncology, Acibadem Healthcare Group, Istanbul 34642, Turkey; 3 Department of Radiation Oncology, Acibadem Research Institute of Senology, Istanbul 34752, Turkey; serap.yucel1@acibadem.com; 4 Department of Breast Radiology, Acibadem University Atakent Hospital, Istanbul 34303, Turkey; irmak.durur@acibadem.com; 5 Department of Medical Oncology, Acibadem University Atakent Hospital, Istanbul 34303, Turkey; mustafa.bozkurt@acibadem.com (M.B.); ali.arican@acibadem.com (A.A.); ibrahim.yildiz@acibadem.edu.tr (I.Y.); 6 Department of General Surgery, Acibadem University Atakent Hospital, Istanbul 34303, Turkey; onur.dulgeroglu@acibadem.edu.tr; 7 Department of General Surgery, Acibadem Research Institute of Senology, Istanbul 34752, Turkey; cihan.uras@acibadem.com

**Keywords:** primary breast surgery, breast cancer, isolated bone metastases, prognosis

## Abstract

**Background**: Breast cancer with isolated bone metastases at initial diagnosis represents a clinically distinct metastatic phenotype, often associated with more indolent biology and relatively favorable outcomes compared with visceral metastatic disease. The survival impact of resecting the intact primary breast tumor in de novo metastatic breast cancer remains controversial. In this study, we evaluated the association between primary breast surgery and overall survival (OS), defined as the time from diagnosis to death attributable to breast cancer, in patients presenting with isolated bone metastatic breast cancer. **Methods**: We performed a retrospective population-based cohort study using the Surveillance, Epidemiology, and End Results (SEER) database. Because the SEER variable for bone metastasis at diagnosis is available from 2010 onward and HER2-defined subtype information is available in the modern SEER era, the effective study period was defined as 2010–2021 rather than the full 2000–2021 SEER release period. Patients with breast cancer and isolated bone metastases at presentation, without evidence of lung, liver, brain, or other distant metastatic sites at diagnosis, were included. Demographic and clinicopathological variables, including age, sex, race, biologic subtype, histology, chemotherapy, radiotherapy, and primary breast surgery, were analyzed. Overall survival (OS), defined as the time from diagnosis to death attributable to breast cancer, was estimated using Kaplan–Meier methods and compared using the log-rank test. Independent prognostic factors were evaluated using multivariable Cox proportional hazards modeling. **Results**: A total of 6500 eligible patients were identified. Surgery of the primary breast tumor was performed in 1513 (23.3%) patients, and 62.8% received chemotherapy. Five-year overall survival (OS) was significantly higher among patients who underwent surgery than among those who did not undergo surgery (59.5% vs. 38.6%; *p* < 0.001). In multivariable analysis, primary breast surgery remained independently associated with improved OS (hazard ratio [HR] 0.54, 95% CI 0.48–0.62; *p* < 0.001). Age, histology, chemotherapy, radiotherapy, and biologic subtype were also associated with prognosis. Sex was not significant in the unadjusted analysis (*p* = 0.188), and the multivariable sex finding was interpreted cautiously because only 96 men were included. **Conclusions**: In this population-based cohort of patients with de novo breast cancer and isolated bone metastases, primary breast surgery was associated with improved survival among selected patients. However, this association should not be interpreted as causal, given the inherent limitations of observational registry data, including treatment selection, potential immortal-time bias, unmeasured metastatic burden, performance status, systemic therapy type and response, and local symptom burden, which are not fully captured in SEER. These findings support careful multidisciplinary consideration of local therapy in selected patients, while emphasizing the need for confirmation in prospectively designed studies.

## Simple Summary

This population-based study explores whether removal of the primary breast tumor is associated with survival outcomes in patients diagnosed with de novo breast cancer and isolated bone metastases. Using the Surveillance, Epidemiology, and End Results (SEER) database, we analyzed 6500 patients identified during the period in which metastatic-site coding at diagnosis was available and observed better survival outcomes among those who underwent primary breast surgery compared with those who did not. This association persisted after adjustment for available demographic and clinicopathological factors. These findings suggest that metastatic breast cancer is not a uniform disease and that patients with bone-only metastases may represent a clinically distinct subgroup in whom individualized multidisciplinary consideration of local therapy may be appropriate. However, because patients selected for surgery are often healthier and may have lower disease burden, better functional status, or a more favorable response to systemic therapy, the results should be interpreted as an association rather than proof of a causal surgical benefit. Therefore, these findings support individualized multidisciplinary discussion, not routine surgery for all patients with de novo metastatic breast cancer, and should be confirmed in prospective studies.

## 1. Introduction

Breast cancer is the most frequently diagnosed malignancy among women and a leading cause of cancer-related mortality worldwide. Metastatic breast cancer (MBC) is biologically heterogeneous, and prognosis varies substantially according to tumor biology, metastatic distribution, and response to systemic therapy. Patients presenting with isolated bone metastases represent a clinically distinct subgroup that can experience more prolonged survival than patients with visceral metastatic patterns. Classical clinical observations showed substantially longer survival when metastatic disease appeared confined to the skeleton, and subsequent studies and reviews confirmed the relatively favorable course of isolated bone disease compared with visceral involvement. This phenotype is often enriched for hormone receptor–positive biology and may remain sensitive to contemporary systemic approaches [1,2,3].

The role of locoregional therapy (LRT) of the intact primary breast tumor in de novo stage IV disease remains controversial. Large observational analyses have repeatedly reported improved survival among patients undergoing primary tumor resection; however, such findings are vulnerable to selection bias and unmeasured confounding [4,5]. Randomized trials have produced mixed results: some trials did not show an overall survival benefit with routine LRT, whereas others suggested a possible long-term survival advantage in selected patients [6,7,8,9]. Differences in eligibility criteria, metastatic burden, systemic therapy standards, and subgroup composition likely contribute to these discrepant outcomes.

Another aspect to consider when discussing locoregional therapy in de novo metastatic breast cancer is the concept of oligometastatic disease. Biological models suggest that a limited number of metastatic sites may represent a transitional state between localized and widely disseminated cancer, raising the possibility that selected patients could benefit from more assertive local strategies. In breast cancer, isolated bone metastasis is frequently viewed within this context, as it is often associated with a more indolent clinical course and longer survival compared with visceral involvement. Emerging evidence from studies focused on oligometastatic breast cancer indicates that carefully chosen patients may experience improved outcomes with combined systemic and local treatment approaches, although clear survival advantages have not been consistently demonstrated [10]. This biological perspective provides additional rationale for exploring the potential role of primary tumor resection in patients presenting with isolated bone metastases.

International guidelines consistently emphasize systemic therapy as the foundation of care for metastatic breast cancer (MBC) and recommend that decisions regarding locoregional therapy (LRT) be individualized, typically after multidisciplinary discussion and with careful attention to patient selection and goals of care [11,12]. Given the frequently indolent natural history of isolated bone metastatic disease, defining prognosis and the potential role of local therapy in this subset remains clinically relevant. In this context, we evaluated survival outcomes and prognostic factors among breast cancer patients presenting with isolated bone metastases using a population-based registry, while specifically acknowledging that observational associations between surgery and survival are vulnerable to selection bias and cannot establish causality.

## 2. Materials and Methods

This research was conducted as a retrospective cohort analysis. Patient data were extracted from the Surveillance, Epidemiology, and End Results (SEER) database of the National Cancer Institute (NCI), and all records were de-identified prior to analysis. The study was carried out in compliance with the principles of the Declaration of Helsinki and adhered to established good clinical practice standards. We obtained data for this study from the US NCI’s SEER-17 registry [November 2023 Sub (2000–2021)]. SEER*Stat program version 9.0.41 was used to extract data. The patient population to be included in the study was determined by selecting from the SEER*Stat program; Site and Morphology Primary Site-labeled = “C50.0-Nipple, C50.1-Central portion of breast, C50.2-Upper-inner quadrant of breast and C50.3-Lower-inner quadrant of breast” AND Stage- Summary/Historic Combined Summary Stage (2004+) = “Distant” AND Extent of Disease SEER Combined Mets at Dx-bone (2010+) = “Yes” AND all other metastatic sites = “No”.

Clinicopathological characteristics, including age, sex, race, ethnicity/origin, tumor location, histology, biologic subtype, surgery type, radiotherapy, and chemotherapy, were extracted from the database when available, and the causes of death were recorded. Age was categorized at 65 years, as this threshold is commonly used in population-based oncology studies to distinguish older adults and to approximate differences in comorbidity burden, treatment tolerance, and competing mortality; additional sensitivity assessments using alternative clinically relevant age categorizations were also considered. Survival analyses were performed according to demographic, clinicopathological, and treatment-related variables. During the assessment of overall survival (OS), the SEER “Survival months” variable was used to define follow-up time, and events were identified as deaths classified as “Dead (attributable to this cancer dx)” according to the SEER cause-specific death classification; patients who were alive or died from other causes were censored. Unknown molecular subtype was retained in descriptive analyses and, in Cox modeling, was handled as a separate/unknown category or evaluated in sensitivity analyses, as specified in the Results. Univariable and multivariable analyses were conducted to identify determinants associated with survival outcomes.

Survival was estimated using the Kaplan–Meier method and compared with log-rank tests. Univariable and multivariable Cox regression analyses were performed, and hazard ratios (HRs) with 95% confidence intervals (CIs) were reported. The multivariable model included available SEER covariates, including age, sex, race, histology, biologic subtype, chemotherapy, radiotherapy, and diagnosis period. Because SEER does not capture several factors that influence surgical selection, the results were interpreted as associations rather than causal effects. A two-sided *p*-value < 0.05 was considered statistically significant.

## 3. Results

### 3.1. Patient Characteristics

A total of 6500 eligible patients were included in the analysis. The cohort was predominantly female (98.5%), and slightly more than half of the patients were younger than 65 years at diagnosis (52.3%). White patients comprised the majority of the study population (78.4%), followed by Black patients (12.2%) and Asian or Pacific Islander patients (7.8%). HR+/HER2− disease was the most common biologic subtype, accounting for 69.5% of cases, whereas triple-negative and HR−/HER2+ tumors were relatively uncommon. Unknown biologic subtype accounted for 10.0% of the cohort and was retained in the descriptive table. Primary breast surgery was performed in 23.3% of patients, chemotherapy was administered in 62.8%, and radiotherapy was administered in 36.2%. Overall, the baseline profile suggests that this cohort was largely composed of patients with hormone receptor-positive disease, consistent with the more indolent clinical course often seen in isolated bone metastatic breast cancer. Baseline distributions are shown in Table 1.

### 3.2. Survival Patterns Across Selected Variables

**Figure 1:**
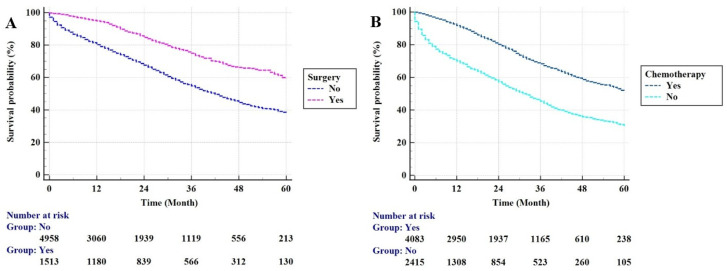
Kaplan–Meier curves for overall survival in breast cancer patients with isolated bone metastases, stratified by primary breast surgery (**A**) and chemotherapy (**B**).

Kaplan–Meier analyses showed clear differences in overall survival according to treatment exposure and tumor-related characteristics. Patients who underwent primary breast surgery had significantly better survival than those who did not, with an estimated 5-year OS of 59.5% versus 38.6%, respectively (log-rank *p* < 0.001) (Figure 1A). A similar survival advantage was observed among patients who received chemotherapy compared with those who did not (Figure 1B). Overall, these findings suggest that both local treatment and systemic therapy were associated with meaningful differences in outcome in this population with isolated bone metastatic disease.

**Figure 2:**
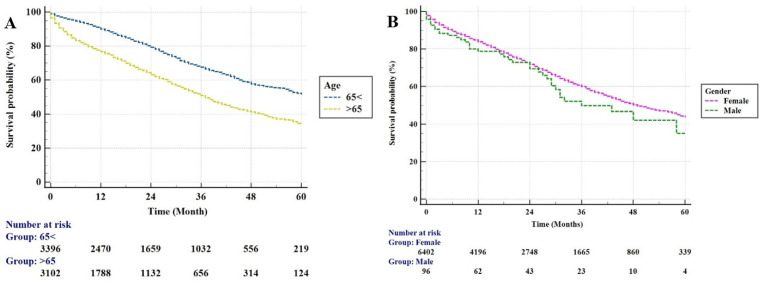
Kaplan–Meier overall survival curves stratified by age (**A**) and sex (**B**) in breast cancer patients with isolated bone metastasis; no significant survival difference was observed by sex, noting the small male sample size.

**Figure 3:**
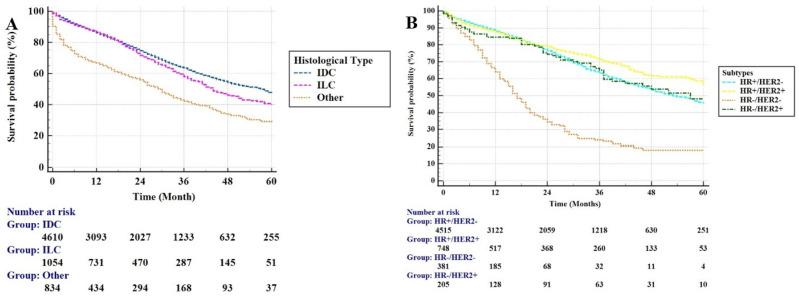
Kaplan–Meier overall survival curves stratified by histological subtypes (**A**), and biologic subtypes (**B**) in breast cancer patients with isolated bone metastases.

Survival differences were also evident across key demographic and disease-specific variables. Younger patients had better overall survival than patients aged 65 years or older (Figure 2A). Sex was not associated with survival in unadjusted Kaplan–Meier analysis, but male sex was associated with worse overall survival in the multivariable model, although this should be interpreted cautiously because of the limited number of male patients (Figure 2B). Additional stratification of survival was observed according to both histologic and biologic subtype (Figure 3A,B), emphasizing the prognostic relevance of both tumor morphology and receptor-defined biology. Overall, the patterns shown in Figure 1, Figure 2 and Figure 3 indicate that isolated bone metastatic breast cancer is not a uniform entity, but rather a clinically distinct subgroup with variable outcomes and a relatively favorable overall prognosis.

### 3.3. Survival Outcomes and Prognosis

In univariable Cox regression analysis, age, race, histologic subtype, biologic subtype, radiotherapy status, chemotherapy status, and primary breast surgery were significantly associated with overall survival. Among these variables, primary breast surgery was associated with a markedly lower risk of death compared with no surgery (HR 0.45, 95% CI 0.40–0.51; *p* < 0.001). Older age was associated with poorer survival (HR 1.85, 95% CI 1.70–2.02; *p* < 0.001), HR−/HER2− disease was associated with markedly worse outcome compared with HR+/HER2− disease (HR 3.18, 95% CI 2.75–3.68; *p* < 0.001), and non-ductal/other histology was also associated with poorer prognosis (HR 2.06, 95% CI 1.84–2.30; *p* < 0.001). These results mirrored the patterns observed in Kaplan–Meier analyses and are presented in Table 2.

On multivariable analysis, primary breast surgery remained independently associated with improved overall survival (HR 0.54, 95% CI 0.48–0.62; *p* < 0.001). Older age likewise retained independent adverse prognostic significance (HR 1.38, 95% CI 1.25–1.52; *p* < 0.001). Tumor-related characteristics remained prognostically relevant, with non-ductal histology and biologic subtype continuing to show significant associations with outcome; notably, HR−/HER2− disease was associated with significantly worse survival relative to HR+/HER2− tumors. Chemotherapy status was also independently associated with survival; however, because SEER chemotherapy coding is limited to yes/no/unknown and does not capture endocrine therapy, HER2-targeted therapy, CDK4/6 inhibitors, antibody–drug conjugates, bone-modifying agents, sequencing, or treatment response, this variable should be interpreted cautiously. Radiotherapy was not significant after adjustment (HR 1.07, 95% CI 0.97–1.18; *p* = 0.150). Taken together, these findings indicate that prognosis in patients with isolated bone metastases reflects treatment exposure, tumor biology, and selection factors, with primary breast surgery representing a strong association rather than evidence of causality (Table 2).

## 4. Discussion

In this population-based cohort of breast cancer presenting with isolated bone metastases, primary breast surgery was associated with improved overall survival in both unadjusted and multivariable analyses. This association is clinically meaningful because isolated bone metastatic disease may follow a more indolent course with longer expected survival than visceral metastatic patterns [1,2,3]. However, the magnitude of the association should be interpreted with caution. In de novo metastatic breast cancer, patients selected for surgery are often younger, fitter, more likely to have controlled systemic disease, lower metastatic burden, fewer symptoms, better access to care, and more favorable tumor biology. These factors are incompletely captured in SEER and may substantially contribute to the observed survival difference. Therefore, our findings should be viewed as hypothesis-generating and associative rather than causal.

The broader randomized evidence for routine resection of the intact primary tumor in de novo MBC remains mixed. The Tata Memorial trial did not demonstrate an overall survival advantage for routine locoregional treatment, with a median OS of 19.2 months in the locoregional treatment group versus 20.5 months in the no-locoregional-treatment group (HR 1.04, 95% CI 0.81–1.34; *p* = 0.79). Similarly, the E2108 trial showed improved locoregional control but no overall survival benefit in patients without progression after initial systemic therapy, with 3-year OS rates of 68.4% in the early locoregional therapy group versus 67.9% in the systemic therapy-alone group (HR 1.11, 90% CI 0.82–1.52; *p* = 0.57) [6,9]. These data argue against a universal recommendation for surgery in all patients with de novo stage IV disease. Conversely, other randomized evidence suggests potential benefit in selected patients. The MF07-01 trial reported a long-term survival difference favoring locoregional therapy, and the ABCSG-28 POSYTIVE trial provides additional prospective data, including quality-of-life considerations, although interpretation across trials is influenced by differences in design, sample size, and systemic therapy era [7,8]. Importantly, observational studies also repeatedly report associations between primary tumor resection and improved survival, but these findings must be interpreted cautiously due to treatment selection and residual confounding [4,5]. Multiple systematic reviews and meta-analyses have attempted to synthesize these conflicting results. Some meta-analyses suggest that LRT may be associated with improved survival in de novo stage IV disease overall, while also highlighting that the pooled benefit is sensitive to study design and that prospective trials alone may not show a statistically significant survival advantage [13,14]. This ongoing debate is echoed in contemporary guidelines, which advocate for individualized consideration of locoregional therapy rather than its routine use in all patients [11,12].

Kaplan–Meier analyses demonstrated clear stratification of overall survival by age, histologic subtype, biologic subtype, and chemotherapy status. Age remains a recognized determinant of outcome in metastatic breast cancer, reflecting treatment tolerance and comorbidity burden [15]. Histologic differences between lobular and ductal carcinoma may influence metastatic behavior and prognosis [16]. Biologic subtype is a major driver of survival, with HR- and HER2-defined groups exhibiting distinct therapeutic responsiveness and outcomes [17]. The survival difference by chemotherapy status likely reflects both treatment effect and patient selection, as systemic therapy remains central in advanced disease management [12]. Sex was not associated with survival in unadjusted analysis; although male sex was associated with worse outcome after multivariable adjustment, this result should be interpreted cautiously because the male subgroup was small and the finding may be unstable [18].

Another relevant consideration is the rapid evolution of systemic therapy over the study period. The introduction of combination endocrine regimens, CDK4/6 inhibitors, more effective HER2-targeted treatments, and antibody–drug conjugates has meaningfully extended survival in metastatic breast cancer, particularly within biologically defined subgroups [19,20]. Therefore, the survival advantage observed among patients undergoing primary surgery may partly reflect improved systemic disease control, treatment selection, and response to therapy, rather than the independent effect of surgery itself [21]. This limitation is particularly important because SEER does not capture endocrine therapy, HER2-targeted therapy, CDK4/6 inhibitors, immunotherapy, antibody–drug conjugates, bone-modifying agents, treatment sequence, or response to induction therapy. It is also especially relevant in this cohort, in which most patients had HR+/HER2− disease, for whom endocrine-based therapy is foundational. Accordingly, the evolving treatment landscape highlights the need to better understand how advances in systemic therapy may interact with locoregional approaches, particularly in biologically favorable settings such as isolated bone metastatic disease [22,23,24].

Overall, our results suggest that primary breast surgery may be linked to improved survival in selected patients with isolated bone metastases, a group that often demonstrates a more indolent clinical course and favorable tumor biology. However, these results should be integrated with randomized trial evidence and contemporary guideline recommendations. The present study should not be interpreted as supporting routine primary tumor surgery for all patients with de novo metastatic breast cancer. Rather, it supports careful multidisciplinary assessment of selected patients, particularly when durable systemic disease control, local symptom prevention, or local disease control are clinically relevant goals [25,26,27,28].

This retrospective registry analysis is subject to treatment selection bias, immortal-time bias, and residual confounding. Important clinical variables such as Eastern Cooperative Oncology Group performance status, comorbidity burden, detailed metastatic burden, number and distribution of bone lesions, oligometastatic versus polymetastatic status, local symptoms, surgery timing, surgical margins, systemic therapy regimen and sequencing, endocrine therapy, HER2-targeted therapy, CDK4/6 inhibitors, antibody–drug conjugates, immunotherapy, bone-modifying agents, and response to systemic treatment are not captured in SEER. Treatment variables are also limited and may be coded as yes/no/unknown. Misclassification and missingness may occur for treatment variables and subtype coding. Although the analysis used a large population-based registry rather than a single-center dataset, registry-based findings remain vulnerable to unmeasured clinical heterogeneity and cannot replace prospective evidence [29,30].

## 5. Conclusions

In this population-based SEER cohort of patients with de novo breast cancer and isolated bone metastases, primary breast surgery was associated with improved survival among selected patients. This association should not be interpreted as causal because treatment selection, unmeasured metastatic burden, performance status, systemic therapy type and response, surgery timing, and local disease symptoms are not fully captured in SEER. The findings support individualized multidisciplinary discussion of locoregional therapy and reinforce the need for prospective studies focused on biologically favorable or oligometastatic subgroups in the modern systemic therapy era [31].

## Figures and Tables

**Table 1:** Baseline Characteristics of the Breast Cancer Patients with Isolated Bone Metastases Included in the SEER Database Between 2010 and 2021.

	Characteristic	Number: 6500	%
**Gender**	Male	96	1.5
	Female	6404	98.5
		6500	100
**Age group (years)**	<65	3398	52.3
	≥65	3102	47.7
		6500	100
**Race**	White	5095	78.4
	Asian or Pacific Islander	510	7.8
	Black	796	12.2
	American Indian/Alaska Native	99	1.5
		6500	100
**Biologic Subtype**	HR+/HER2−	4515	69.5
	HR+/HER2+	748	11.5
	HR−/HER2−	381	5.9
	HR−/HER2+	205	3.2
	Unknown	651	10.0
		6500	100
**Primary Breast Surgery**	Yes	1513	23.3
	No	4960	76.3
	Unknown	27	0.4
		6500	100
**Radiotherapy**	Yes	2350	36.2
	No	4044	62.2
	Unknown	106	1.6
		6500	100
**Chemotherapy**	Yes	4083	62.8
	No/Unknown	2417	37.2
		6500	100

**Table 2:** Univariable and Multivariable Cox Regression Analysis of Factors Associated with Overall Survival in Patients With Isolated Bone Metastases Included in the SEER Database Between 2010 and 2021.

	Univariate Analysis		Multivariate Analysis	
	Hazard Ratio for OS (95% CI)	*p*-Value	Hazard Ratio for OS (95% CI)	*p*-Value
Age <65 years ≥65 years	Reference 1.85 (1.70–2.02)	<0.001	Reference 1.38 (1.25–1.52)	<0.001
Sex Female Male	Reference 1.23 (0.90–1.69)	0.188	Reference 1.43 (1.03–1.99)	0.031
Primary Breast Surgery No Yes	Reference 0.45 (0.40–0.51)	<0.001	Reference 0.54 (0.48–0.62)	<0.001
Race White Black Asian or Pacific Islander American Indian/Alaska Native	Reference 1.30 (1.15–1.47) 0.75 (0.63–0.89) 0.87 (0.51–1.48)	<0.001 0.002 0.624	Reference 1.30 (1.13–1.49) 0.85 (0.70–1.02) 1.19 (0.69–2.06)	<0.001 0.092 0.526
Origin Spanish–Hispanic–Latino Non-Spanish–Hispanic–Latino	Reference 1.06 (0.93–1.21)	0.334	Reference 0.94 (0.82–1.09)	0.461
Tumor Type IDC ILC Other	Reference 1.15 (1.02–1.29) 2.06 (1.84–2.30)	0.015 <0.001	Reference 1.10 (0.97–1.24) 1.21 (1.04–1.40)	0.112 0.012
Molecular subtype HoR+/HER2− HoR+/HER2+ HoR−/HER2− HoR−/HER2+	Reference 0.79 (0.68–0.92) 3.18 (2.75–3.68) 1.04 (0.81–1.34)	0.002 <0.001 0.732	Reference 0.97 (0.83–1.13) 4.05 (3.48–4.72) 1.56 (1.20–2.01)	0.736 <0.001 0.001
Radiotherapy Yes No/Unknown	Reference 1.39 (1.27–1.52)	<0.001	Reference 1.07 (0.97–1.18)	0.150
Chemotherapy Yes No/Unknown	Reference 2.14 (1.63–2.81)	<0.001	Reference 1.99 (1.80–2.19)	<0.001

Multivariate analysis model *p*-value < 0.001, Abbreviations: CI, confidence interval; HoR, hormone receptor; HER2, human epidermal growth factor receptor 2.

## Data Availability

The datasets generated and/or analyzed during the current study are available in the Surveillance, Epidemiology, and End Results (SEER) Program repository, https://seer.cancer.gov/data-software/ (accessed on 15 June 2025).
